# Examining the Utility of a Sleep Resource in Transdiagnostic Internet-Delivered Cognitive Behavior Therapy: An Observational Study

**DOI:** 10.3390/ijerph19159337

**Published:** 2022-07-30

**Authors:** Vanessa Peynenburg, Andreea Ababei, Andrew Wilhelms, Michael Edmonds, Nick Titov, Blake F. Dear, Viktor Kaldo, Susanna Jernelöv, Heather D. Hadjistavropoulos

**Affiliations:** 1 Department of Psychology, University of Regina, 3737 Wascana Parkway, Regina, SK S4S 0A2, Canada; vanessa.peynenburg@uregina.ca (V.P.); aaf439@uregina.ca (A.A.); andrew.wilhelms@uregina.ca (A.W.); michael.edmonds@uregina.ca (M.E.); 2 MindSpot Clinic, School of Psychological Sciences, Macquarie University, Sydney, NSW 2109, Australia; nick.titov@mq.edu.au; 3 eCentreClinic, School of Psychological Sciences, Macquarie University, Sydney, NSW 2109, Australia; blake.dear@mq.edu.au; 4 Centre for Psychiatry Research, Department of Clinical Neuroscience, Karolinska Institutet, & Stockholm Health Care Services, Region Stockholm, SE 14186 Stockholm, Sweden; viktor.kaldo@ki.se (V.K.); susanna.jernelov@ki.se (S.J.); 5 Department of Psychology, Faculty of Health and Life Sciences, Linnaeus University, SE 35195 Vaxjo, Sweden; 6 Division of Psychology, Department of Clinical Neuroscience, Karolinska Institutet, SE 17177 Stockholm, Sweden

**Keywords:** insomnia, transdiagnostic, internet-delivered cognitive behavior therapy

## Abstract

Patients seeking transdiagnostic internet-delivered cognitive behavior therapy (T-ICBT) for anxiety or depression often have sleep difficulties. A brief resource that includes sleep psychoeducation and strategies for improving sleep (e.g., stimulus control and sleep restriction) may address comorbid insomnia without the need for an insomnia-specific ICBT course. This observational study explored patient use and feedback of a brief sleep resource available to all patients (*n* = 763) enrolled in an 8-week T-ICBT course. Overall, 30.1% of patients (*n* = 230) reviewed the resource and were older, more engaged with the ICBT course (i.e., more likely to complete the program, more logins, and greater number of days enrolled in the course) and had higher pretreatment insomnia symptoms than those who did not review the resource. Resource reviewers did not report larger improvements in symptoms of insomnia than non-reviewers, even among patients with clinical levels of insomnia, and average insomnia levels remained above the clinical cutoff at posttreatment. While patients were satisfied with the resource and it was beneficial to some patients, more research is needed to further explore how it may be integrated into T-ICBT and how therapists can encourage the use of the resource among patients who may benefit from the resource.

## 1. Introduction

Transdiagnostic internet-delivered cognitive behavior therapy represents a valuable approach for improving patient access to care [1]. In this approach, patients access carefully developed web-based lessons or modules that provide psychoeducation and support people to learn and use broadly relevant cognitive and behavioral skills. The internet-delivered approach improves the convenience of care (e.g., access in any location and at any time), while the transdiagnostic approach addresses the high rates of comorbidity found in routine care settings. From the patients’ perspective, transdiagnostic approaches can be beneficial because they allow for significant improvements across a number of clinical concerns without having to engage in several courses of therapy [2]. For therapists, transdiagnostic approaches reduce the need for training in multiple disorder-specific protocols and have been found to be as effective in reducing symptoms of depression and anxiety in ICBT [3]. Transdiagnostic internet-delivered cognitive behavior therapy (T-ICBT) is both an acceptable and effective alternative to face-to-face CBT [4].

In transdiagnostic ICBT, the focus is typically on depression and anxiety and includes content that mirrors traditional CBT (e.g., cognitive restructuring, breathing or relaxation strategies, behavioral activation, graded exposure, and relapse prevention; see Reference [5]). During a T-ICBT intervention, patients also often access extra materials that include psychoeducational content and skills for specific issues and difficulties that are expected to be relevant for some, but not all, patients [6]. Sleep represents one such problem area that is relevant for many patients and is likely important to address in T-ICBT. Patients who experience major depressive disorder or anxiety disorders often have comorbid sleep difficulties [7], and patients who complete CBT for depression and anxiety often still have elevated sleep problems after treatment [8,9,10]. Sleep problems such as insomnia have been associated with an increased risk for developing medical and psychiatric disorders [11,12], and a bidirectional relationship has been found between insomnia and depression [13]. In a population-based prospective study examining the relationship between insomnia and depression [14], individuals who had insomnia at baseline were at an increased risk of developing major depressive disorder (OR: 6.2), and those with major depressive disorder at baseline were at an increased risk of developing insomnia (OR: 6.7). In the case of anxiety disorders, there is a moderate lifetime association between anxiety and insomnia, and anxiety disorders have been found to precede insomnia in over 70% of cases of comorbidity [15]. Insomnia has also been associated with extensive personal and societal costs, including missed work, lost productivity, and increased use of healthcare services (see Reference [16]). The combined findings that sleep problems are prevalent and disabling conditions that can contribute to the onset of additional psychiatric concerns highlight the potential value of transdiagnostic approaches.

To date, limited attention has been given to sleep within the context of T-ICBT. One previous analysis of T-ICBT patients [17] found that 73.4% of patients (339/462) reported sleep problems (e.g., difficulty initiating or maintaining sleep). In that study, an item analysis was conducted, examining mean scores on the sleep concern question of the Patient Health Questionnaire 9 (PHQ-9; [18]) at pretreatment and posttreatment. A 46% reduction in scores on the sleep concern question was found, suggesting that T-ICBT appears to be associated with reduced sleep concerns, even when sleep is not directly targeted in the intervention. However, one major limitation of this study is that it relied on a single item from the PHQ-9 instead of a validated sleep measure. This study also did not examine patient use and evaluations of a sleep resource. Answers to these questions could help inform improvements to T-ICBT as well as assist therapists who are delivering T-ICBT, particularly concerning patients with comorbid sleep difficulties. 

The primary objective of this study was to examine the uptake and utility of including a resource on improving sleep within an 8-week T-ICBT program for anxiety and depression. A secondary objective was to collect descriptive data to inform how to best integrate information about improving sleep in T-ICBT. In this observational utility study, we were interested in examining the following: (1) the percentage of patients who make use of a sleep resource, (2) factors associated with the use of a sleep resource, (3) extent of symptom change from pretreatment to posttreatment among users of a resource, and (4) patients’ evaluations of the sleep resource. This study was exploratory in nature, so no hypotheses were made regarding the percentage of patients that would access the resource or what patients’ evaluations of the resource would entail. It was hypothesized, however, that patients screening positive for a likely insomnia diagnosis would be more likely to access the resource. Finally, we hypothesized that patients who accessed the sleep resource would have larger decreases in insomnia than those who did not access the sleep resource, especially among patients who are likely to actually suffer from insomnia.

## 2. Materials and Methods

### 2.1. Design and Ethics

This study used the data collected as part of the Online Therapy Unit’s regular service delivery. The Research Ethics Board at the University of Regina approved the use of data for research purposes (REB file: 2019-197).

### 2.2. Participants

#### 2.2.1. Recruitment

Patients learned about the Online Therapy Unit and ICBT from various sources, including community mental health clinics, medical professionals, word of mouth, and online searches. Other referrals sources include the media, email announcements, and posters and cards [19]. Patients must first apply for ICBT through the Online Therapy Unit which is a government-funded clinic that provides ICBT to Saskatchewan residents. 

#### 2.2.2. Sample Size

This study included all patients (*N* = 763) who began ICBT between 4 January 2021 and 5 July 2021. A 6-month time span was chosen to allow for an evaluation of the Good Sleep Resource usage trends. Using G-power, we estimated that a sample size of 352 would be sufficient to detect a small between-group effect size (0.30) in insomnia improvement, using *t*-tests between reviewers and non-reviewers of the resource. 

#### 2.2.3. Eligibility Criteria

To be eligible for ICBT and, thus, the current study, patients had to report that they were (a) at least 18 years old; (b) experiencing at least mild symptoms of anxiety or depression as primary concerns on the measures listed below; (c) able to access and comfortable using a computer and the internet; (d) living in Saskatchewan for the 8-week treatment; (e) able to provide a medical contact in the event of an emergency; and (f) interested in, and willing to consent to, ICBT at the time of screening. 

Patients were excluded from the current study if they (a) were experiencing severe alcohol or drug problems, (b) had unmanaged psychosis or mania, (c) were assessed as being at high suicide risk, and (d) were receiving mental health services from another provider more than twice a month.

### 2.3. Measures

Although patients complete various measures as part of the *Wellbeing Course*, the measures listed below are the focus of the current study. As part of the screening process, patients’ demographic information was obtained. Data are only described for those patients who were accepted into the trial and completed the consent form for treatment. At screening and posttreatment (8 weeks), the Insomnia Severity Index (ISI; [20]), Patient Health Questionnaire 9 (PHQ-9; [18]), Generalized Anxiety Disorder 7 (GAD-7; [21]), and Sheehan Disability Scale (SDS; [22]) were administered. Lastly, at posttreatment, the Treatment Satisfaction Questionnaire and Sleep Resource Evaluation Survey were administered. 

#### 2.3.1. Demographics

Demographic characteristics were collected during screening, including age, gender, marital status, education, employment status, ethnicity, and location.

#### 2.3.2. ISI

The ISI is a 7-item self-report questionnaire that asks about insomnia symptoms such as difficulties falling asleep, staying asleep, and early waking. Items are rated on a scale from “0” (not at all) to “4” (extremely), with a total score ranging from 0 to 28 [20]. The ISI has been identified as having good reliability and validity in Canadian samples, and a score of 10 or more has been found to be optimal for detecting cases of insomnia [23]. Cronbach’s alpha in the current study ranged from 0.88 to 0.90.

#### 2.3.3. PHQ-9

The PHQ-9 consists of 9 items and measures depressive symptoms [18]. Respondents rate items on a scale from “0” (not at all) to “3” (nearly every day), with a total score ranging from 0 to 27 [18]. The PHQ-9 is commonly used as a screening measure for depression. A score of less than 5 is indicative of minimal depression, while a score of 10 or more has been use as a cutoff score for a probable diagnosis of major depression [24]. The PHQ-9 has been validated in Canadian samples and has good psychometric properties [25]. Cronbach’s alpha in the current study ranged from to 0.83 to 0.88.

#### 2.3.4. GAD-7

The GAD-7 is a 7-item validated self-report questionnaire that measures the acuteness of anxiety symptoms [21], with good psychometric properties in Canadian samples [26]. Patients rate how often items have bothered them in the last 2 weeks, from “0” (not at all) to “3” (nearly every day), producing a total score ranging from 0 to 21. Minimal anxiety is indicated by a score of less than 5, whereas clinically severe anxiety symptoms are indicated by a score of 10 or more [21]. Cronbach’s alpha in the current study ranged from to 0.86 to 0.90.

#### 2.3.5. SDS

The SDS is a 3-item measure that asks respondents to rate the impairment of their functioning in three domains: work/school, social life, and family life [22]. Items are scored on a scale from “0” (not at all) to “10” (extremely). The psychometric properties have been established in the literature [22]; however, they have not been reported specifically in a Canadian sample. Cronbach’s alpha in the current study ranged from to 0.81 to 0.89.

#### 2.3.6. Treatment Satisfaction Questionnaire

At posttreatment, patients were asked to rate how satisfied they were with the overall treatment on a 5-point scale (1 = “very dissatisfied”; 5 = “very satisfied”). Following that, they were asked if the course was worth their time (“yes” or “no”) and if they would recommend the course to a friend (“yes” or “no”). Patients also rated the impact of the course on their confidence in managing symptoms, as well as their motivation to seek treatment in the future on a 5-point scale (1 = “greatly reduced”; 5 = “greatly increased”).

#### 2.3.7. Sleep Resource Evaluation Survey

All quantitative and qualitative data about patients’ use of and experiences with the Good Sleep Resource were collected from the Sleep Resource Evaluation Survey. At posttreatment, patients were asked whether they reviewed the Good Sleep Resource during the course (“yes” or “no”), and if so, how much effort they put into working on the resource on a 7-point scale (1 = “none at all”; 7 = “a great deal”). Using a scale from 1 (“not at all”) to 7 (“very”), patients rated how understandable the resource was, if they learned something new by reviewing the resource, and how helpful the resource was. Lastly, patients were encouraged to provide feedback on what they liked and disliked about the Good Sleep Resource, as well as describe any changes to how they managed sleep based on reviewing the resource.

#### 2.3.8. Engagement

Various aspects of treatment engagement were measured, including patients’ preference for optional versus standard weekly therapist contact (described in more detail below), the number of lessons accessed, emails sent to therapist, emails received from therapist, phone calls with the therapist, logins to the website, and the number of days between the first and last login to the website. 

### 2.4. Intervention

#### 2.4.1. The Wellbeing Course

The current study used an ICBT coursed named the *Wellbeing Course*. This course was developed by researchers at the eCentreClinic at Macquarie University (MQU) in Sydney, Australia (for further information, see Reference [27]), and licensed by the Online Therapy Unit. The *Wellbeing Course* is an 8-week transdiagnostic intervention for symptoms of anxiety disorders and major depressive disorders. The course contains five online lessons that cover (1) symptom identification and the cognitive behavioral model, (2) thought monitoring and challenging, (3) de-arousal strategies and pleasant activity scheduling, (4) graduated exposure, and (5) relapse prevention [27]. Each lesson contains psychoeducational information in the form of presentation-like slides, case stories, and a downloadable guide that includes homework assignments and frequently asked questions to help with skill acquisition. Over the course of eight weeks, materials were gradually released, with patients receiving regular automated emails advising them of the availability and content of the next lesson [28]. Consistent with past research [28], patients had access to additional resources on a number of topics, including assertiveness, communication skills, managing beliefs, managing worry, mental skills, managing panic, posttraumatic stress disorder, sleep, managing alcohol use, workplace mental health, emergency information, managing anger, balancing new motherhood, chronic conditions, chronic pain, COVID-19, grief, health anxiety, and motivation, that are available at any time. As with the other additional resources, patients could access the Good Sleep Resource at any point during the intervention. During the first message to patients, therapists discussed the availability of these resources and made tailored recommendations for resources the patients should review based on their presenting concerns. Therapists then followed up anytime patients mentioned the use of a resource. Furthermore, at week five, therapists asked patients if they had any questions regarding the resources.

#### 2.4.2. Good Sleep Resource

The resource consists of 14 pages and includes 4 sections that target different aspects of sleep. The first section introduces patients to general information about sleep and insomnia. Patients then learn about various factors (e.g., biological, psychological, behavioral, environmental, and social) affecting sleep. The subsequent section introduces a variety of skills for better sleep that patients are encouraged to engage in. The skills are derived mostly from CBT for insomnia [29], which has a solid evidence base to improve insomnia severity [30] and include skills and strategies such as managing thoughts and beliefs associated with sleep, developing bedtime routines, and engaging in stimulus control and sleep restriction therapy; it also include skills for managing sleep for shift workers. For stimulus control, patients are encouraged to limit all non-sleep activities (except sex) in the bedroom to enhance the association between sleepiness and the bedroom, while also removing cues for arousal that are induced by stimulating activities such as eating, using a smartphone, or watching TV in bed. As part of sleep restriction, patients are encouraged to sleep on a strict schedule (e.g., limiting sleeping in and napping) beginning with minor sleep deprivation to assist patients with adjusting their circadian rhythm to feel sleepy at bedtime and alert during wake time. Patients are provided with a recommended timeline to proceed through each stage of sleep restriction. As part of sleep restriction, a printable sleep diary is provided that patients may use to track their sleep and rate the quality of their sleep. Patients are prompted to start by tracking their sleep over the span of a week, using the diary, which includes ratings for fatigue (1–10), naps (hours), the time they went to bed, the time they fell asleep, wake-up time, total sleep time, and sleep quality (1–10). The final section provides answers to the most frequently asked questions about sleep and summarizes strategies for improving the severity of insomnia symptoms. The resource was initially developed by MQU and then expanded upon by collaborating with researchers who have developed and evaluated CBT for insomnia, for instance, in the form of guided self-help CBT for insomnia [8,31,32]. A patient-oriented research committee consisting of patients, managers, and e-therapists was also involved in the review of the material before implementation.

#### 2.4.3. Therapist Support

Patients were assigned one therapist for the duration of the ICBT course and provided either optional or regular once-weekly support, which are both found to be effective for delivering ICBT (see Reference [33] for additional details on the optional and standard therapist support). Specifically, patients who scored in the clinical range on either the GAD-7 or PHQ-9 could pick between regular weekly support or optional weekly support. Patients who scored in the non-clinical range on both the anxiety and depression measures (<10 on the PHQ-9 and GAD-7) were automatically offered optional weekly support. With regular weekly support, therapists reviewed patient questionnaires and messages once a week and then provided patients a tailored brief message sent via secure messaging on the website. If clinically indicated, therapists also contracted patients by phone (i.e., patient had not logged in for a week, patient’s score on the GAD-7 or PHQ-9 increased by 5 or more points, or patient’s message or questionnaires indicated elevated suicide risk). In each message, therapists were expected to include certain details related to patient symptom measures and progress in the course (see Reference [34] for more details). In optional support, therapists only contacted patients who initiated contact or if therapists were concerned about patient safety after examining their weekly symptom questionnaires (i.e., increase of 5 or more points on the GAD-7 or PHQ-9, or elevated suicide risk on the PHQ-9).

### 2.5. Analyses

During a preliminary review of the data, three groups emerged: patients who reported accessing the Good Sleep Resource (reviewers), patients who reported they did not access the Good Sleep Resource (non-reviewers), and patients who did not respond to the questionnaire (QNR). Descriptive statistics were used to describe the demographic and clinical characteristics of these three groups. One-way ANOVAs and chi-square tests were used to compare for pretreatment differences among the three groups. 

Next, change scores were calculated for the ISI, PHQ-9, GAD-7, and SDS, with negative values indicating a decrease in symptom severity from pretreatment to posttreatment. One-way ANOVAs were then conducted to assess whether reviewers and non-reviewers differed in regard to change scores, and *t*-tests were used to examine group differences on continuous measures of treatment engagement and satisfaction. Comparisons of different methods for estimating treatment effect (i.e., ANOVA, analysis of covariance, and linear mixed modeling) yield similar treatment effect estimates [35], so an ANOVA was chosen for comparing change scores. An additional one-way ANOVA was conducted to assess whether resource reviewers with clinical levels of insomnia (ISI > 10) had larger change scores on the ISI than non-reviewers did. Within-group effect sizes (Cohen’s d) were computed for changes on each of the primary measures from pretreatment to posttreatment. Chi-square tests were used to assess whether reviewers and non-reviewers differed on categorical measures of engagement and satisfaction. It was not possible to include the QNR group in these analyses since the QNR group did not provide posttreatment or satisfaction data. A *p*-value of 0.01 was considered statistically significant as a partial control for multiple comparisons. 

Patient feedback regarding the Good Sleep Resource was examined by using a conventional qualitative content analysis approach [36] due to the brief length of patient responses and the ability to quantify the data into different categories of responses [37]. The qualitative analysis was completed in three steps:

1. After six months of offering the *Wellbeing Course*, the authors analyzed responses at posttreatment from 230 patients who reviewed the Good Sleep Resource. Initial coding was completed by A.A. and A.W., where A.W. examined the last 50 responses and A.A. examined the remaining responses for each open-ended question. Each author independently identified broad categories.
2. A.A. and A.W. then met to discuss thoughts of the initial categories created. They created a coding guide with code classifications and descriptions for categories and subcategories. All responses were coded by A.A., using the coding guide. Following that, A.W. reviewed the coding and then met with A.A. to discuss any discrepancies.
3. An experienced coder and co-author (H.D.H.) examined the responses to adjust and confirm categories and to resolve any disagreements in assigned codes.

More complex patient responses could be assigned more than one code (e.g., if they described liking more than one aspect of the Good Sleep Resource).

## 3. Results

### 3.1. Patient Characteristics

The patient demographic characteristics at pretreatment are shown in Table 1. The mean age of all patients was 37.67 years (SD = 13.98), with a range between 18 to 84 years. Most patients were white (86.6%; *n* = 661), 77.2% (*n* = 589) were female, 59.2% (*n* = 452) were married/common-law, 44.4% (*n* = 339) reported completing more than high school/less than university, 50.2% (*n* = 383) were employed part-time or full-time, and 57.7% (*n* = 440) lived in a large city (>100,000 residents). Lastly, over half (54.9%; *n* = 419) of patients reported using psychotropic medication in the last 3 months.

There were several pretreatment differences between the three groups (reviewers, non-reviewers, and QNR). The reviewers of the *Good Sleep Resource* were significantly older than those in the other two groups (*p* < 0.001 in both cases). Fewer reviewers were single compared to the QNR group (27.9% vs. 39.0%; χ^2^ (1, 505) = 7.04; *p* = 0.008). Furthermore, there were more reviewers who were homemakers, students, or retired compared to the QNR group (33.0% vs. 22.6%; χ^2^ (1, 763) = 6.25; *p* = 0.01). Significantly more reviewers were of white ethnicity compared to the QNR group (91.7% vs. 82.5%; χ^2^ (1, 505) = 6.62; *p* = 0.01). Finally, there were more non-reviewers with university education than in the QNR group (38.4% vs. 26.9%; χ^2^ (1, 533) = 9.64; *p* = 0.002). 

When looking at pretreatment symptom severity, 72.3% (*n* = 522) of patients had a score ≥ 10 on the ISI suggestive of insomnia, 73.3% (*n* = 559) had clinical levels of depression (PHQ-9 ≥ 10), and 72.0% (*n* = 549) had clinical levels of generalized anxiety (GAD-7 ≥ 10). Only 9.0% (*n* = 69) of patients did not have any scores in the clinical ranges of either anxiety, depression, or insomnia. The only statistically significant difference in the proportion of patients in the clinical range at pretreatment was that reviewers were more likely to score above the cutoff for insomnia than non-reviewers (79.6% vs. 60.5%; χ^2^ (1, 763) = 27.78; *p* < 0.001) (see Table 1).

### 3.2. Symptom Changes from Pretreatment to Posttreatment

Table 2 provides information about symptom-change scores for reviewers and non-reviewers on the ISI, PHQ-9, GAD-7, and SDS. As seen in Table 2, there was a statistically significant pretreatment difference between the two groups, whereby reviewers had a higher pretreatment ISI mean compared to non-reviewers. There were no statistically significant group differences for any of the change scores *(p* range: 0.07 to 0.41). A sub-analysis comparing ISI change scores between resource reviewers and non-reviewers with clinical levels of insomnia was not statistically significant (*F* (1, 337) = 0.16; *p* = 0.69).

Table 3 includes within-group Cohen’s d effect sizes for primary measures among resource reviewers and non-reviewers, as well as among resource reviewers and non-reviewers with ISI scores ≥ 10.

### 3.3. Engagement

Patient treatment engagement for reviewers and non-reviewers is found in Table 4. Overall, 58.4% (*n* = 285/488) of patients received optional once-weekly support, while 45.3% (*n* = 203/488) of patients received standard once-weekly support. When looking at treatment completion across the reviewers and non-reviewers, 94.1% (*n* = 459/488) accessed lesson four, while 87.7% (*n* = 428/488) accessed lesson five, and 99.8% (*n* = 487/488) completed the primary measures at posttreatment. In terms of differences in engagement, reviewers were more likely to access lesson five compared to non-reviewers (93.0% vs. 82.9%; χ^2^ (1, 488) = 11.47; *p* < 0.001). Overall, patients logged into the course an average of 26.65 times (SD = 22.52), with an average of 81.81 (SD = 32.54) days between first and last login. Throughout the treatment, patients sent an average of 3.46 (SD = 3.02) messages to their therapist, received an average of 7.09 (SD = 2.64) messages from their therapist, and had an average of 0.93 (SD = 1.30) phone calls with their therapist. Reviewers had significantly more logins compared to non-reviewers (M = 29.67, SD = 29.29 vs. M = 23.96, SD = 13.45; *F* (1, 486) = 7.95; *p* = 0.005) and a greater number of average of days between first and last login (M = 85.90, SD = 33.40 vs. M = 78.17, SD = 21.38; *F* (1, 486) = 6.95; *p* = 0.009).

### 3.4. Treatment Satisfaction

Table 4 also displays patient treatment satisfaction for reviewers and non-reviewers. Most patients (81.4%; *n* = 397/488) were either satisfied or very satisfied with the overall treatment. Nearly all patients reported that the treatment was worth their time (96.3%; *n* = 470/488) and that they would recommend the course to a friend (96.1%; *n* = 469/488). Furthermore, 83.8% (*n* = 409/488) of patients felt that the treatment either increased or greatly increased their confidence in their ability to manage their symptoms, and 77.9% (*n* = 380/488) of patients indicated that the course either increased or greatly increased their motivation to seek additional help, if necessary, in the future. No significant differences were found between reviewers and non-reviewers on measures of satisfaction (*p* range: 0.35 to 0.98).

### 3.5. Evaluation of the Good Sleep Resource

The average amount of effort patients placed into the resource was 4.76 (SD = 1.52) on a 7-point scale (1 = “none at all”; 7 = “a great deal”). Patients found the resource to be very understandable (M = 6.11, SD = 1.04) on a scale ranging from 1 (“not at all”) to 7 (“very”). Patients also gave the resource an average rating of 4.71 (SD = 1.81) for learning something new from it and 4.96 (SD = 1.65) for how helpful it was, both using a scale from 1 (“not at all”) to 7 (“very”). Lastly, nearly all patients (92.1%; *n* = 210/228) reported that the resource was worth their time.

### 3.6. Qualitative Feedback about the Good Sleep Resource

#### 3.6.1. Liked Course Aspects

Patients reported liking several aspects of the Good Sleep Resource (see Table 5). The most commonly liked aspect of the resource was the overall information it provided (*n* = 87/230; 37.8%), followed by psychoeducation about sleep (*n* = 84/230; 36.5%). Sleep improvement strategies accounted for 24.3% of responses (*n* = 56/230), which were broken down into developing a bedtime routine (11.7%; *n* = 27/230), stimulus control (7.0%; *n* = 16/230), and sleep restriction (7.0%; *n* = 16/230). Other patients (15.2%; *n* = 35/230) liked the presentation of the resource (e.g., format, structure, layout, and ability to download). Only 12.1% (*n* = 28/230) of patients did not report anything they liked about the resource and 3.5% (*n* = 8/230) reported that the content did not apply to them (i.e., have no issues with sleep).

#### 3.6.2. Disliked Sleep Resource Aspects

Table 6 includes the disliked aspects of the Good Sleep Resource. Of note, when asked about dislikes, the majority of patients either reported enjoying the resource content (48%; *n* = 111/230) or did not provide feedback about what they disliked (27.0%; *n* = 62/230). Some patients indicated that the resource did not provide new information (13.0%; *n* = 30/230). Others thought that the content of the resource was not relevant to their experiences, such that they wanted additional information or they found the content unhelpful (11.7%; *n* = 27/230), or that they had issues with the format of the resource, such as wanting more personal support from their therapist (1.3%; *n* = 3/230). Some comments suggested adding information to the resource about over-sleeping (“I struggle with sleeping too much and I don’t recall any part of [the *Good Sleep Resource*] having to do with over-sleeping”^33964^), the relationship between trauma and insomnia, and managing shift-work schedules (“Maybe I’m expecting there to be some sort of solution of going from day shifts to night shifts, but a heavier focus on that would be helpful”^34400^).

#### 3.6.3. Changes Made to Sleep

Table 7 provides a summary of the changes patients made to how they managed sleep after reviewing the resource. Overall, 58.7% (*n* = 135/230) of patients reported that they made changes to managing sleep, 51.3% (*n* = 118/230) said that it is a work in progress, 34.3% (*n* = 79/230) reported that they did not make changes, and 4.8% said that they did not know (*n* = 11/230). Among those who made changes, the most common change was developing bedtime routines (37%; *n* = 85/230), followed by stimulus control (17.8%; *n* = 41/230), and sleep restriction therapy (15.7%; *n* = 36/230).

## 4. Discussion

The current study explored the utility of a sleep resource that was available to patients throughout an 8-week transdiagnostic ICBT course. Study objectives included examining the percentage of patients who accessed the resource, pretreatment characteristics of resource reviewers, whether reviewers and non-reviewers report different changes in symptoms (insomnia, depression, anxiety, and mental-health-related disability) from pretreatment to posttreatment, patient engagement and satisfaction of reviewers and non-reviewers, and how patients evaluated the Good Sleep Resource. Furthermore, the study included an analysis of open-ended feedback about what patients liked and disliked about the resource and if they changed their sleep habits. It was hypothesized that patients with more severe levels of insomnia at pretreatment would be more likely to review the resource and that reviewers would have larger change scores on the ISI from pretreatment to posttreatment. No further hypotheses were made given the exploratory nature of this study.

### 4.1. Utilization of Resource

Overall, 30.1% of patients (*n* = 230/763) reported reviewing the Good Sleep Resource during the T-ICBT course. Of note, 36.0% of patients (*n* = 275/763) did not respond to the survey, and therefore it is unknown whether they reviewed the resource or not. Clinical levels of insomnia symptoms were reported in 72.3% of all patients, and thus less than half of patients who would likely benefit from the sleep resource actually reviewed it. Within the current study, patients who had more severe sleep problems were more likely to review the resource, but more effort could potentially be put forth to ensure that the resource is utilized by all patients experiencing clinical levels of sleep problems. Patients who are seeking ICBT for depression and/or anxiety may not be aware of the bidirectional relationship between their mental health symptoms and sleep problems, so therapists may need to provide psychoeducation about the bidirectional relationship early in treatment for patients to understand the rationale for accessing the sleep resource.

### 4.2. Characteristics of Resource Reviewers

There were several pretreatment differences between reviewers and non-reviewers. The finding that reviewers were more likely to score in the clinical range on the ISI and had higher mean ISI scores at pretreatment compared to non-reviewers was consistent with our hypotheses. Resource reviewers were also older, and this finding is consistent with research on predictors of ICBT patient outcomes, which have found that older patients are more likely to complete ICBT (see Reference [38]). Within the literature on CBT for insomnia, it has also been found that older age is associated with a greater likelihood of attending at least three sessions, as well as better adherence to sleep schedules [39].

Resource reviewers did not report statistically significant larger reductions in ISI scores than non-reviewers. An additional analysis that compared ISI change scores between resource reviewers and non-reviewers who had clinical levels of insomnia also did not reach significance. These null findings suggest that the sleep resource was not enough on its own to contribute to significant improvements in sleep beyond the significant improvements experienced as a result of the T-ICBT course. A likely explanation is that patients who reviewed the resource did not use the most important methods of sleep restriction and stimulus control enough (as suggested by patient comments). Furthermore, therapists focused primarily on the core content of the T-ICBT course in their messages and only offered support regarding the sleep resource if patients discussed the resource in their messages. Reviewers did not have significantly larger reductions in insomnia symptoms, even among those with clinical levels of insomnia, and the fact that reviewers had higher pretreatment ISI scores complicates direct comparison. An alternative explanation for this null finding is that both reviewers and non-reviewers experienced improvements in their sleep as a result of the T-ICBT course and that our analyses were not sensitive to any between-group differences associated with review of the Good Sleep Resource. This is, however, contradicted by previous findings that ICBT for depression, given to patients with both depression and insomnia, did not reduce insomnia symptoms but only symptoms of depression, while ICBT for insomnia reduced both types of symptoms significantly also after three years [8,40]. In T-ICBT, patients are encouraged to apply the core skills to their specific areas of concern, so patients in the current study may have applied skills such as thought challenging and de-arousal strategies to their symptoms of insomnia and experienced a decrease in symptoms without reviewing the Good Sleep Resource. In a meta-analysis of CBT-I [41], effect sizes of 0.5 (95% CI = 0.3, 0.8) were reported for reduction of insomnia severity in patients who had comorbid insomnia and depression. Among patients who scored in the clinical range for insomnia symptoms, we found that resource reviewers (Cohen’s d = 0.88; 95% CI = 0.70, 1.05) and non-reviewers (Cohen’s d = 0.80; 95% CI = 0.62, 0.98) had larger effect sizes for insomnia reductions than reported in the meta-analysis, thus suggesting that the T-ICBT course likely had more of an impact on insomnia symptoms than the sleep resource did. The observational nature of this study limits our ability to draw conclusions about the possible additive benefits of reviewing the resource in addition to using the core skills to address sleep concerns. Given that reviewers had more severe symptoms of insomnia both pre- and posttreatment, it may be that reviewers included a subgroup of individuals with sleep problems that are not likely to be solved through the use of the CBT-based techniques covered in the Good Sleep Resource (e.g., chronic health conditions and life circumstances that cannot currently be changed). Still, the finding that mean scores on the ISI remained above the clinical cutoff among resource reviewers suggests that there remains room to improve the effectiveness of the resource in reducing symptoms of insomnia.

No specific hypotheses were made regarding the differences in program engagement between reviewers and non-reviewers. There were significant group differences in engagement, whereby reviewers were more likely to complete all five lessons of the T-ICBT program (93.0% vs. 82.9%), logged onto the treatment platform a greater number of times (M = 29.67, SD = 29.29 vs. M = 23.96, SD = 13.45), and had a greater number of days between their first and last login (M = 85.90, SD = 33.40 vs. M = 78.17, SD = 21.38). Resource reviewers were more engaged in the T-ICBT course across several different metrics, so this sub-group of patients may comprise treatment “doers” [42]. In a qualitative analysis of patients’ engagement with ICBT, “doers” were defined as patients who take a practice-oriented approach to treatment, apply the material to their real-life experiences, can overcome obstacles in their practice of skills, appreciate taking ownership of the treatment experience, and have positive attitudes toward ICBT. In the current study, treatment “doers” likely benefitted from the combination of being highly engaged with the core content, as well as reviewing the materials, and implementing skills from the additional resources they could access throughout the course.

### 4.3. Patient Feedback on the Resource

The qualitative content analysis of patient responses revealed several suggestions that can guide the future development of the *Good Sleep Resource,* such as providing additional information about how patients’ comorbid health concerns or work schedules impact their sleep. Patients reported liking the general knowledge the resource provided and psychoeducation about sleep the most. Patients also particularly enjoyed learning about sleep methods such as sleep restriction, stimulus control, and bedtime routines. The finding that patients enjoyed learning about sleep restriction suggests that patients may not have actually engaged in sleep restriction, as patients often report that sleep restriction is challenging to engage in [43]. The nature of patients’ responses to the Sleep Resource Evaluation Survey limits our interpretation of what aspects patients liked the most about the resource, as it is unclear whether they liked reading about the skills or enjoyed actually practicing the skills. Only a very small percentage of patients identified aspects of the resource that they disliked (e.g., lack of new information), and some patients identified specific topics that they would like to see addressed in the resource (e.g., hypersomnia, posttraumatic stress disorder and insomnia, and more information about shift work). Most patients indicated that they made changes to how they manage sleep, including implementing sleep restriction, stimulus control, and a bedtime routine. Patients also commonly reported that managing sleep is still a work in progress, and a small number of patients stated that they did not make any modifications to their sleeping habits.

The findings from this study are promising for several reasons. First, we found that patients who had more severe insomnia symptoms at pretreatment were more likely to report accessing the Good Sleep Resource, thus suggesting that the patients who would likely derive the most benefit from the resource are the ones accessing it. Second, 58.7% (*n* = 135/230) of patients reported that they had made changes to their sleep as a result of reading the resource. Although there was no direct measurement of patients’ engagement with the resource, this finding suggests that more than half of patients made changes that were substantial enough to recall at posttreatment, and it is possible that other patients made more short-term changes. Third, patients were satisfied with the resource overall, with 92.1% of patients (*n* = 210/228) reporting that the resource was worth their time. Taken together, the findings of this study suggest that including a resource on managing sleep problems will appeal to a target audience of patients who struggle with insomnia, that reviewers of the resource will make meaningful changes to their sleep, and that the resource is perceived as acceptable to patients. These findings are important from an implementation perspective because they suggest that a good portion of patients are using and benefitting from the Good Sleep Resource.

### 4.4. Limitations and Future Directions

The nature of the resource evaluation survey administration, treatment platform, and observational study resulted in several limitations that provide opportunities for future research directions. First, the resource evaluation survey was administered at posttreatment at the same time that patients completed primary outcome measures. Only 63.9% (*n* = 488/763) of patients completed the resource evaluation survey. A limitation in the treatment platform’s metrics meant that we had to rely solely on patients’ self-reports of whether they accessed the resource, and data were missing for over one-third of patients, so it is possible that a larger group of patients did review the Good Sleep Resource. If we had obtained data from all 763 patients, then our analyses comparing reviewers and non-reviewers would have had more power to detect between-group differences. Alternatively, relying on self-reports may have been more meaningful than using the website’s metrics, as some patients may download resources without reading them, and it is impossible to know the extent of engagement a patient had with the resource based on website metrics alone. In future studies, it may be worthwhile to include a resource evaluation survey earlier in the treatment (e.g., mid-treatment or triggered one week after reviewing the resource) to ensure higher completion rates. Approximately 60% of patients who did not review the resource had ISI scores above the clinical cutoff, so it appears that a substantial group of patients who might have benefitted from the resource did not access it. Patients may have been overwhelmed by the list of additional resources that they could choose from and decided that the sleep resource was not a priority. For patients whose symptoms of depression or insomnia include difficulties with focus or concentration, they might have chosen to focus their efforts on the five core lessons instead of the additional resources. Additionally, some patients might not perceive their sleep concerns as a causal factor in their distress and may therefore have not accessed the sleep resource. In the future, it may be worthwhile asking non-reviewers about their reasons for not accessing the resource.

Future studies could use a randomized controlled trial design in which all patients who score in the clinical range on the ISI are randomized to either receive the sleep resource or not. Different methods of offering information about sleep strategies could also be explored, such as offering the information as an additional resource (as in the current study) compared to including a whole T-ICBT lesson on insomnia within the course. Additionally, for patients who have a probable insomnia diagnosis, therapists in the future could better use ISI scores to monitor symptom improvement and direct patient attention to the most effective methods of improving insomnia (i.e., stimulus control and sleep restriction) and then follow up with patients on these strategies during the treatment period. Patients’ responses to the open-ended survey questions were brief, so it might be beneficial to invite patients to complete semi-structured interviews over the phone to gain a better understanding of their likes and dislikes, as well as any changes they made after reviewing the resource. Over two-thirds of patients did not access the sleep resource, but it is unclear whether they did not access it because they did not perceive it as worthwhile, that they did not have any significant sleep concerns, or if there is another explanation for not accessing the resource. It is possible that patients felt that the core T-ICBT course was helpful enough in reducing their symptoms of insomnia and therefore saw minimal value in accessing the resource. An additional limitation of this is the reliance on the ISI as an indicator of insomnia rather than a formal diagnosis of insomnia. Furthermore, symptoms of insomnia were only measured at pretreatment and posttreatment, so we are unable to draw conclusions about the potential long-term impacts of reading the resource.

In general, it is important to note that patients who participated in this study may have had greater treatment motivation than observed in other routine care settings, in that all patients actively sought out and applied for treatment. There was also a lack of diversity in the sample in terms of gender and ethnicity, with most patients identifying as female (77.2%, *n* = 589/763) and white (86.6%, *n* = 661/763). While it is the case that the prevalence of insomnia is higher among females than males [44], the results of this study may not be generalizable to patients who do not identify as female. Furthermore, there is some evidence that ethnicity factors into insomnia trajectory [45], so future studies should aim to include patients from different ethnicities to ensure that the sleep resource is beneficial for those groups as well.

### 4.5. Strengths

Some research suggests that patients with comorbid depression and insomnia who receive an ICBT program for insomnia experience larger improvements in both symptom areas compared to those who received an ICBT program for depression [40]. This finding illustrates the importance of tailoring ICBT to address comorbid concerns, particularly for patients with insomnia. To the best of our knowledge, previous studies have not examined how including a sleep resource in a pre-existing T-ICBT can impact patients with comorbid insomnia and anxiety/depression. The current study contributes to the literature by providing evidence for the utility of a resource that patients can access at any point during T-ICBT, which may be beneficial for patients with primary symptoms of anxiety and depression who are hoping to improve their sleep. A further strength of this study is the inclusion of both objective (e.g., lesson completion) and subjective measures (e.g., open-ended evaluations) of patient engagement, outcomes, and experiences with the Good Sleep Resource. The information gathered in this study about the use, content, and evaluation of the Good Sleep Resource is critical to determining its place in routine ICBT. The study included feedback from patients about the Good Sleep Resource, which may help to inform future changes to the resource, such as how the sleep strategies can be applied or modified for patients with comorbid health concerns or shift work. Furthermore, qualitative feedback about patients’ unmet needs related to sleep can help inform therapists’ practices when working with patients who have comorbid insomnia. Patients’ feedback also suggests that the most important sleep strategies (i.e., sleep restriction and stimulus control) were not the most commonly used skills, so greater emphasis on the importance of these strategies may be necessary.

## 5. Conclusions

Many patients who seek ICBT for anxiety or depression also experience symptoms of insomnia. One approach to address comorbid insomnia is by including a brief resource that provides psychoeducation on sleep, strategies for improving sleep (e.g., challenging thoughts related to sleep), and information on sleep restriction and stimulus control. Overall, we found support for the inclusion of a sleep resource in T-ICBT. While less than a third of patients reported reviewing the resource, it did seem to appeal to the target audience of patients with higher pretreatment insomnia. Resource reviewers had higher initial levels of insomnia symptoms, were older, and more engaged with the overall T-ICBT program (i.e., more likely to complete treatment, greater number of logins, and greater number of days enrolled in the program) compared to patients who did not review the resource. Change scores on the measure of insomnia were comparable between resource reviewers and non-reviewers and were statistically significant in both cases. Patients were satisfied with the sleep resource, and most of them reported making changes to their bedtime routines and habits as a result of reading the resource, but the most demanding and most effective techniques in CBT for insomnia, sleep restriction and stimulus control, were clearly under-utilized. Patients also offered suggestions for how the resource can be improved for future studies, such as including information on how to improve sleep for patients who work night shifts or have specific medical concerns. Overall, the findings of this study suggest that patients with insomnia perceive a sleep resource as an acceptable and helpful addition to T-ICBT and that future research should explore ways to increase patient engagement with the most important therapeutic techniques to reduce sleep problems for those with more severe insomnia symptoms.

## Figures and Tables

**Table 1:** Patient characteristics at pretreatment.

Variable	All Patients (*N* = 763)		Accessed Good Sleep Resource (Reviewers) (*n* = 230)		Did Not Access Good Sleep Resource (Non-Reviewers) (*n* = 258)		Questionnaire Non-Responders (QNR) (*n* = 275)		Statistical Significance
	*n*	%	*n*	%	*n*	%	*n*	%	
Age									
Mean (SD)	37.67 (13.8)	-	43.93 (15.28)	-	35.68 (12.85)	-	34.32 (12.07)	-	*F* (2, 760) = 36.72; *p* = 0.001
Range	18–84	-	18–81	-	18–84	-	18–77	-	
Gender									
Female	589	77.2	176	76.5	205	79.5	208	75.6	χ^2^ (1, 763) = 0.04; *p* = 0.84
Male/Other	174	22.8	54	23.5	53	20.5	67	24.4	
Marital status									
Single/never married	251	32.9	64	27.9	80	31.0	107	39.0	χ^2^ (1, 763) = 7.36; *p* = 0.007
Married/common-law	452	59.2	143	62.2	160	62.0	149	54.1	
Separated/ divorced/widowed	60	7.9	23	10.0	18	7.0	19	6.9	
Education									
High school or less	163	21.4	50	21.7	44	17.1	69	25.1	χ^2^ (1, 763) = 5.55; *p* = 0.02
More than high school/ less than university	339	44.4	92	40.0	115	44.6	132	48.0	
University education	261	34.2	88	38.3	99	38.4	74	26.9	
Employment status									
Employed part-time/full-time	383	50.2	104	45.2	131	50.8	148	53.8	χ^2^ (1, 763) = 6.25; *p* = 0.01
Unemployed/disability	153	20.1	50	21.7	38	14.7	65	23.6	
Homemaker/Student/ retired	227	29.8	76	33.0	89	34.5	62	22.6	
Ethnicity									
White	661	86.6	211	91.7	223	86.4	227	82.5	χ^2^ (1, 763) = 6.32; *p* = 0.012
Indigenous	45	5.9	7	3.0	14	5.4	24	8.7	
Other	57	7.5	12	5.2	21	8.1	24	8.7	
Location									
Large city (over 100,000)	440	57.7	133	57.8	149	57.8	158	57.5	χ^2^ (1, 763) = 0.28; *p* = 0.59
Small to medium city	113	14.8	30	13.0	34	13.2	49	17.8	
Small rural location (under 10,000)	210	27.5	67	29.1	75	29.1	68	24.7	
Pretreatment scores									
Insomnia Severity Index ≥10	522	72.3	183	79.6	156	60.5	213	77.5	χ^2^ (1, 763) = 27.78; *p* < 0.001
Pretreatment PHQ-9 ≥ 10	559	73.3	174	75.7	164	63.6	221	80.4	χ^2^ (1, 763) = 2.00; *p* = 0.16
Pretreatment GAD-7 ≥ 10	549	72.0	159	69.1	177	68.8	213	77.5	χ^2^ (1, 763) = 4.61; *p* = 0.03
No clinical scores	69	9.0	17	7.4	33	12.8	19	6.9	χ^2^ (1, 763) = 6.69; *p* = 0.04
Psychotropic medication in the past 3 months	419	54.9	127	55.2	127	49.2	165	60.0	χ^2^ (1, 763) = 1.42; *p* = 0.23
Pretreatment credibility Mean (SD)	20.70 (4.54)	-	20.70 (4.29)	-	20.62 (4.55)	-	20.77 (4.75)	-	*F* (2, 760) = 0.07; *p* = 0.94

Notes: PHQ-9 = Patient Health Questionnaire 9; GAD-7 = Generalized Anxiety Disorder 7.

**Table 2:** Pretreatment and posttreatment symptom scores and change scores.

Variable (Post *n*)	Combined (*n* = 488)			Accessed Good Sleep Resource (Reviewers) (*n* = 230)			Did Not Access Good Sleep Resource (Non-Reviewers) (*n* = 257–258)			Statistical Significance (Pretreatment Differences)	Statistical Significance (Pre-Post Change Scores)
	Pre-Mean	Post-Mean	Change Score	Pre-Mean	Post-Mean	Change Score	Pre-Mean	Post-Mean	Change Score		
ISI	13.16 (6.49)	9.82 (6.20)	−3.32 (5.36)	14.95 (6.25)	11.14 (6.31)	−3.80 (5.46)	11.57 (6.29)	8.63 (5.85)	−2.92 (5.25)	*F*(1, 486) = 35.32; *p* < 0.001	*F* (1, 485) = 3.30; *p* = 0.07
PHQ-9	12.69 (5.51)	6.79 (5.24)	−5.88 (5.40)	13.27 (5.30)	7.05 (5.36)	−6.22 (5.74)	12.17 (5.65)	6.56 (5.12)	−5.58 (5.06)	*F* (1, 486) = 4.80; *p* = 0.03	*F* (1, 485) = 17.72; *p* = 0.19
GAD-7	12.09 (4.97)	6.18 (4.91)	−5.89 (5.22)	12.24 (5.10)	6.03 (4.89)	−6.21 (5.47)	11.96 (4.86)	6.32 (4.94)	−5.61 (4.98)	*F* (1, 486) = 0.40; *p* = 0.53	*F* (1, 485) = 1.61; *p* = 0.21
SDS	17.41 (7.26)	13.79 (8.13)	−3.62 (7.88)	17.67 (7.82)	14.36 (8.34)	−3.31 (8.10)	17.19 (6.74)	13.29 (7.92)	−3.90 (7.68)	*F* (1, 486) = 0.53; *p* = 0.47	*F* (1, 485) = 0.68; *p* = 0.41

Notes: ISI = Insomnia Severity Index; PHQ-9 = Patient Health Questionnaire 9; GAD-7 = Generalized Anxiety Disorder 7; SDS = Sheehan Disability Scale.

**Table 3:** Effect sizes (Cohen’s d, 95% CI) for symptom changes from pretreatment to posttreatment.

Variable	Overall Sample (*n* = 488)		Patients with Clinical Levels of Insomnia (*n* = 339)	
	Accessed Good Sleep Resource (Reviewers) (*n* = 230)	Did Not Access Good Sleep Resource (Non-Reviewers) (*n* = 257–258)	Accessed Good Sleep Resource (Reviewers) (*n* = 183)	Did Not Access Good Sleep Resource (Non-Reviewers) (*n* = 156)
	Cohen’s d (95% CI)	Cohen’s d (95% CI)	Cohen’s d (95% CI)	Cohen’s *d* (95% CI)
ISI	0.70 (0.56, 0.84)	0.56 (0.43, 0.69)	0.88 (0.70, 1.05)	0.80 (0.62, 0.98)
PHQ-9	1.08 (0.92, 1.25)	1.10 (0.95, 126)	1.13 (0.95, 1.32)	1.19 (0.99, 1.40)
GAD-7	1.14 (0.97, 1.30)	1.13 (0.97, 1.28)	1.17 (0.98, 1.36)	1.14 (0.93, 1.34)
SDS	0.41 (0.27, 0.54)	0.51 (0.38, 0.64)	0.43 (0.28, 0.58)	0.49 (0.33, 0.66)

Notes: ISI = Insomnia Severity Index; PHQ-9 = Patient Health Questionnaire 9; GAD-7 = Generalized Anxiety Disorder 7; SDS = Sheehan Disability Scale.

**Table 4:** Treatment engagement and satisfaction.

Variable	Combined		Accessed Good Sleep Resource (Reviewers)		Did Not Access Good Sleep Resource (Non-Reviewers)		Statistical Significance
	(*n* = 488)		(*n* = 230)		(*n* = 258)		
	*n*	%	*n*	%	*n*	%	
Support							
Optional 1x week	285	58.4	134	58.2	151	58.5	χ^2^ (1, 488) = 0.004; *p* = 0.95
Standard 1x week	203	41.6	96	41.7	107	41.5	
Engagement							
Accessed lesson 4	459	94.1	222	96.5	237	91.9	χ^2^ (1, 488) = 4.72;
							*p* = 0.03
Accessed lesson 5	428	87.7	214	93	214	82.9	χ^2^ (1, 488) = 11.47; *p* < 0.001
Completion of 8-week primary measures	487	99.8	230	100	257	99.6	χ^2^ (1, 488) = 0.89;
							*p* = 0.35
Mean written messages received from therapist (SD)	7.09	-	7.3	-	6.90 (2.66)	-	*t* (486) = 1.71;
	−2.64		−2.61				*p* = 0.09
Mean written messages sent to therapist (SD)	3.46	-	3.79	-	3.17 (2.60)	-	*t* (486) = 2.29;
	−3.02		−3.39				*p* = 0.02
Mean number of logins (SD)	26.65	-	29.67 (29.29)	-	23.96 (13.45)	-	*t*(486) = 2.82;
	−22.52						*p* = 0.005
Mean number of phone calls with therapist (SD)		-	0.83	-	1.02 (1.40)	-	*t* (486) = 1.57;
	0.93		−1.17				*p* = 0.12
	−1.3						
Mean days between first and last login (SD)	81.81	-	85.90 (33.40)	-	78.17 (21.38)	-	*t* (486) = 2.64;
	−32.54						*p* = 0.009
Satisfaction							
Satisfied/very satisfied overall	397	81.4	185	80.4	212	82.2	χ^2^ (1, 488) = 0.24;
							*p* = 0.62
Course was worth the time (%)	470	96.3	222	96.5	248	96.1	χ^2^ (1, 488) = 0.05;
							*p* = 0.82
Would recommend course to friend (%)	469	96.1	221	96.1	248	96.1	χ^2^ (1, 488) = 0.00;
							*p* = 0.98
Increased/greatly increased confidence	409	83.8	189	82.2	220	85.3	χ^2^ (1, 488) = 0.86;
							*p* = 0.35
Increased/greatly increased motivation for other treatment	380	77.9	182	79.1	198	76.7	χ^2^ (1, 488) = 0.40;
							*p* = 0.53

**Table 5:** Patient responses to the question, what did you like about the resource? (*n* = 230).

Liked	Example	Patient ID	*n*	%
Overall information (positive feedback, everything, general tips for sleep)	“I liked how informative it was and quite relatable to be able to help some of my insomnia needs”.	34048	87	37.8
Sleep education (learning new things, reinforce, remind)	“It reminded me that healthy sleep is an important determinant of overall health and that I should focus on making my sleep healthy as well”.	33995	84	36.5
Sleep improvement strategies	“It gave me strategies to use to develop a good sleep routine/develop better sleep habits”.	35000	56	24.3
Sleep restriction	“It gave me a plan that WORKED to help me sleep. It gave me confidence to stay awake and wake up early to feel tired at night”.	35198	16	7.0
Stimulus control	“Had to learn that was not a place to hang out. It was for peace and sleep. No phone or movies”.	33902	16	7.0
Routine	“The advice about creating a healthy bedtime routine that starts with doing something relaxing in the evening before even going to bed”.	33952	27	11.7
Presentation (format, structure, layout, downloadable)	“Everything was well laid out and easy to understand”.	34390	35	15.2
Nothing/can’t remember (N/A, no response, not sure)	“Nothing stands out in my mind”.	34256	28	12.1
Content doesn’t apply (no issue sleeping)	“The information was there to use but I do not have a problem sleeping”.	34772	8	3.5

Note: Responses could be coded more than once.

**Table 6:** Patient responses to question, what did you not like about the resource? (*n* = 230).

Disliked	Example	Patient ID	*n*	%
Nothing/ found all content helpful	“Everything was great about this course”.	34769	111	48.3
N/A, not sure, don’t remember, no response	“N/A”.	33949	62	27.0
No new information	“There wasn’t a lot of new information to me”.	33947	30	13.0
Not relevant to patient experiences (wanting additional information, unhelpful content)	“I was looking for more information on managing sleep when working night shifts. The resource touched on it minimally and I did not find it helpful”.	34764	27	11.7
Format issues	“My attention span for reading right now is not so good so I wished I could watch a video instead but I managed”.	34560	3	1.3

Note: Responses could be coded more than once.

**Table 7:** Patient responses to question, did you make any changes to how you manage sleep based on the *Good Sleep Resource*? (*n* = 230).

Changes Made	Example	Patient ID	*n*	%
Yes	“Yes. I focus more on getting a good sleep”.	34046	135	58.7
Sleep restriction	“I used the sleep restriction strategy to set up a stricter sleep schedule. I would tend to stay up later than I should some nights, so I now make sure that I always go to bed at the same time”.	35264	36	15.7
Stimulus control	“Yes. Bedroom is now distraction free. No TV or technology”.	34544	41	17.8
Routine	“I started to try and exercise more, and ‘wind-down’ by trying to do more bedtime yoga stretches before bed, or reading a book to limit screen time. I also started practicing controlled breathing in bed to help me relax”.	33866	85	37.0
Work in progress/trying	“I’ve tried. Sleep is an ongoing battle, but I am working on heathy sleep habits”.	33864	118	51.3
No	“No changes”.	34852	79	34.3
I don’t know, N/A	“N/A”.	33858	11	4.8

Note: Responses could be coded more than once.

## Data Availability

The data presented in this study are available upon request from the corresponding author.
